# An Integrated Human/Murine Transcriptome and Pathway Approach To Identify Prenatal Treatments For Down Syndrome

**DOI:** 10.1038/srep32353

**Published:** 2016-09-02

**Authors:** Faycal Guedj, Jeroen LA Pennings, Lauren J. Massingham, Heather C. Wick, Ashley E. Siegel, Umadevi Tantravahi, Diana W. Bianchi

**Affiliations:** 1 Mother Infant Research Institute, Tufts Medical Center and the Floating Hospital for Children, Boston, MA, United States; 2 Center for Health Protection (GZB), National Institute for Public Health and the Environment (RIVM), Bilthoven, The Netherlands; 3 Department of Computer Science, Tufts University, Medford, MA, United States; 4 Department of Pathology, Women and Infants’ Hospital, Providence, RI, United States

## Abstract

Anatomical and functional brain abnormalities begin during fetal life in Down syndrome (DS). We hypothesize that novel prenatal treatments can be identified by targeting signaling pathways that are consistently perturbed in cell types/tissues obtained from human fetuses with DS and mouse embryos. We analyzed transcriptome data from fetuses with trisomy 21, age and sex-matched euploid controls, and embryonic day 15.5 forebrains from Ts1Cje, Ts65Dn, and Dp16 mice. The new datasets were compared to other publicly available datasets from humans with DS. We used the human Connectivity Map (CMap) database and created a murine adaptation to identify FDA-approved drugs that can rescue affected pathways. *USP16* and *TTC3* were dysregulated in all affected human cells and two mouse models. DS-associated pathway abnormalities were either the result of gene dosage specific effects or the consequence of a global cell stress response with activation of compensatory mechanisms. CMap analyses identified 56 molecules with high predictive scores to rescue abnormal gene expression in both species. Our novel integrated human/murine systems biology approach identified commonly dysregulated genes and pathways. This can help to prioritize therapeutic molecules on which to further test safety and efficacy. Additional studies in human cells are ongoing prior to pre-clinical prenatal treatment in mice.

The intellectual disability in Down syndrome (DS) is associated with delays in neurogenesis, synaptogenesis, and brain growth that first become apparent during fetal development^1^. Few efforts, however, have focused on developing prenatal therapies that can improve embryonic brain development and neonatal cognitive outcomes in DS. To date, human clinical trials have been mainly conducted on adolescents and young adults in whom processes that are critical for brain development and cognition are largely complete^2^. Our laboratory is focused on identifying and testing treatments that a pregnant woman can take orally following a prenatal diagnosis of trisomy 21 (T21). We hypothesize that therapies that specifically target signaling pathways and cellular processes that are perturbed in fetuses with DS will positively impact brain growth and wiring, leading to significant improvement of postnatal cognitive outcome^3^.

We previously analyzed cell-free fetal RNA (cffRNA) from the amniotic fluid (AF) of living human fetuses diagnosed with T21 and age and sex-matched euploid controls to compare real-time gene expression abnormalities that occur *in utero*^4^. Our findings suggested that fetuses with DS have genome-wide gene expression changes that affect multiple cellular processes and signaling pathways, including oxidative stress, ion transport, cell stress response and G-protein signaling^4^,^5^. The use of cffRNA has, however, two major limitations: (1) the transcripts in the amniotic fluid are partly degraded and provide limited information compared to other human tissues, and (2) cffRNA from AF does not fully reflect brain development even though it contains several brain specific transcripts^6^.

To overcome these challenges, we developed a novel integrated human/murine systems biology approach by analyzing the transcriptome of multiple cell types and tissues from both humans with DS and mouse models. This included a newly generated dataset of gene expression in cultured human fetal amniocytes as well as a reanalysis of published datasets in induced pluripotent stem cells (iPSCs) and iPSCs-derived neurons^7^ and post-mortem human fetal brain tissue (cerebellum and cerebrum)^8^,^9^. We also generated new expression data using embryonic forebrains from the three most commonly used mouse models of DS (Ts1Cje, Ts65Dn and Dp16) and compared the mouse and human transcriptomes. Using these integrated analyses, we established a list of signaling pathways and cellular processes that can be further evaluated as candidates for prenatal treatment. We used the Connectivity map (www.broadinstitute.org/CMap) database for humans and adapted it to the mouse to test whether maternal treatment induces therapeutic effects in the pups. In contrast to other drug screening databases that contain only a limited drug library, the Connectivity Map (CMap) database contains more than 7,000 gene expression profiles generated before and after treatment of five different cell lines (MCF7, ssMCF7, HL60, PC3, SKMEL) with 1,309 FDA-approved compounds. It uses unbiased pattern-matching algorithms to identify drugs that can counteract an abnormal transcriptome signature in a disease state^10^,^11^.

Our goal was to use an objective transcriptome based approach, and to interrogate the CMap database for unbiased identification of FDA-approved molecules that have a rational basis for therapy, and can be further evaluated for safety and efficacy in preclinical trials.

## Materials and Methods

### Sources of RNA

#### Human Amniocytes

This study was approved by the Institutional Review Boards at Tufts Medical Center and Women and Infants’ Hospital (Tufts protocol ID: 5582, Women and Infants’ protocol ID: 01-0028). All methods were carried out in accordance with the approved guidelines after obtaining informed consent from all pregnant women participating in this study. Second trimester amniocytes were obtained after centrifugation at 1,100 RPM for 5 min from residual second trimester AF samples of women carrying singleton fetuses undergoing clinically indicated genetic testing. The initial sample set consisted of fourteen flasks of amniocytes with the following metaphase karyotypes: 47, XX, +21 (*N* = 4); 47, XY, +21 (*N* = 3); 46, XX (*N* = 4); 46, XY (*N* = 3). Gestational ages ranged from 15 2/7 to 20 5/7 weeks. Amniocytes (10^+5^ cells) were plated in 25 cm^2^ cell culture flasks and incubated at 37 °C (20% O_2_, 5% CO_2_) until they reached 90–95% confluence. The optimized AmnioMax™ C-100 basal media and AmnioMax™ C-100 supplement (Life Technologies, Grand Island, NY) were used for culture. For RNA extraction amniocyte density was adjusted to 10^5^ cells/ml (10 ml total volume) and centrifuged at 1100 RPM for five min. The cell pellet (10^6^ cells) was rinsed with ice-cold PBS 1X before proceeding to RNA extraction. RNA was extracted using TRIzol^**®**^ Reagent according to the manufacturer’s instructions (Qiagen Inc, Valencia, CA). RNA concentrations were measured as absorbance at 260 nm and RNA quality was analyzed with the Bioanalyzer (Agilent Biotechnologies, Santa Clara, CA).

#### Mouse Embryonic Forebrain

All murine experiments were conducted according to international ethical standards detailed in the World Medical Association Declaration of Helsinki and approved by the Institutional Animal Care and Use Committee (IACUC) of Tufts University (Protocol B2013-20). Dp16 (B6-Dp(16Lipi-Zfp295)1Yey/J; Stock: 013530) and Ts1Cje males (B6-T(12;16)1Cje/CjeDnJ) were crossed with C57Bl/6J females (Jackson Laboratories, Bar Harbor, ME). Ts65Dn females (B6EiC3Sn.BLiA-Ts(1716)65Dn/DnJ; Stock: 005252) were crossed with C57BL/6JEi × C3H/HeSnJ F1 hybrid (B6EiC3) males. The presence of a vaginal plug was defined as embryonic day 0.5 (E0.5) and 10% weight gain at embryonic day 10 (Johnson *et al*. 2010). Embryos were recovered at E15.5 and the forebrain was dissected before storage at −80 °C^12^. Total RNA was isolated from the developing forebrain of Dp16 (N = 6), Ts65D (N = 5), Ts1Cje (N = 5) embryos and their respective euploid littermates (N = 6, 6 and 5 respectively) using the RNA II kits, following the manufacturer’s instructions (Macherey-Nagel, Bethlehem, PA).

### Identification of Differentially Regulated Genes in Humans with DS and Mouse Models

#### Human Amniocytes

RNA of amniocytes derived from seven fetuses with trisomy 21 (T21) and sex and gestational age-matched euploid fetuses was converted to cDNA and hybridized to Affymetrix Human U133 Plus 2.0 arrays (Affymetrix, Santa Clara, CA) as described previously^4^. Fourteen arrays were used (7 T21 and 7 euploid); each array corresponded to labeled cDNA from one amniocyte culture. Analyses were performed using a paired t-test and Benjamini-Hochberg False Discovery Rates (BH-FDR) of 5% and 20% as a cut-off^13^. We used paired t-tests to match for fetal sex and gestational age.

#### Mouse models embryonic day 15.5 forebrain

RNA was converted to cDNA and hybridized to Affymetrix mouse gene 1.0 ST arrays (Affymetrix, Santa Clara, CA) as described previously^12^. Twenty-three arrays were used to generate this dataset (6 Dp16 and 6 WT littermates, 5 Ts65Dn and 6 WT littermates); each array corresponded to labeled RNA from one individual embryonic forebrain. The gene expression data from the Ts1Cje embryonic forebrain were published previously (NCBI GEO ID: GSE62538). For each mouse strain, sex-genotyping was performed using *Sry* targeted primers (*Sry-F*: GCTGGGATGCAGGTGGAAAA; *Sry-R*: TGATGGCATGTGGGTTCCTG) as described previously^13^. Equal numbers of male and female embryos were used in all experiments.

Statistical analyses were carried out on the normalized data using R software (version 2.13.1). Gene expression data from Dp16, Ts65Dn and Ts1Cje tissues were compared to their respective control littermates using an unpaired t-test and Benjamini-Hochberg cut-offs of 5% and 20%. Ts1Cje, Ts65Dn and Dp16 mouse models carry duplications of 77, 128 and 145 protein and non-protein-coding genes on the mouse chromosome 16, respectively.

### Identification of Pathway Abnormalities in Humans with DS and Mouse Models

#### Human Cells and Tissues From Individuals with DS

Functional pathway analyses were performed using the Gene Set Enrichment Analysis (GSEA) with the Developmental FunctionaL Annotation at Tufts (DFLAT), the Database for Annotation, Visualization, and Integrated Discovery (DAVID) and Ingenuity Pathway Analysis (Qiagen, Redwood City, CA)^14^,^15^,^16^. We compared the amniocyte dataset with our previously published data from the second trimester AF supernatant (NCBI GEO ID: GSE16176), and datasets generated by other groups using iPSCs and iPSC-derived neurons (NCBI GEO IDs: GSE48611, GSE42956) as well as human post-mortem fetal cerebrum and cerebellum data (NCBI GEO ID: GSE1397) from fetuses with T21 and age and sex-matched euploid fetuses^4^,^7^,^9^,^17^. Because of the limited number of differentially-regulated genes detected at FDRs off 5% and 20%, we used the top 1% up- and down-regulated genes in all of these datasets for a more comprehensive functional pathway analysis.

#### Embryonic Forebrain in Mouse Models of DS

We used the same functional analytic databases and approaches to investigate pathway similarities and differences in Ts1Cje, Ts65Dn and Dp16 embryonic forebrains.

#### Comparison of Pathway Abnormalities in Humans and Mice

We performed comparative analyses for the human cells and tissues and the mouse embryonic forebrain transcriptomes using GSEA/DFLAT, DAVID and IPA to identify pathway changes that were consistently dysregulated in the different species.

### Screening for Molecules That Target Commonly Dysregulated Pathways

Probe sets that were statistically-significantly differentially up and down-regulated were used to query the CMap Database to identify molecules that show opposite gene expression responses. Potentially therapeutic molecules will have a negative enrichment score on a scale between 0 and −1.0. Because the CMap database uses exclusively human Affymetrix probe set identifiers, the murine gene sets had to be converted to the corresponding human Affymetrix probe set IDs. To accomplish this, murine genes were first converted to their human homolog (using an R algorithm and information from NCBI’s HomoloGene) and subsequently matched to Affymetrix U133A Plus 2.0 probe sets (using probe set annotation data downloaded from the Affymetrix website). Because of the limited number of differentially regulated genes at FDR 20%, we uploaded the list of top 1% up- and down-regulated human probes and the top 1% up- and down-regulated “humanized” murine probes to the human and murine-adapted CMaps.

To screen for relevant drugs that can reverse abnormal transcriptome signatures in both humans with DS and in mouse models, we ranked the CMap drug output for each cell type and tissue based on their enrichment scores and used a cut-off value of −0.7 to select the most relevant molecules. We performed a quality control check on the list of drugs proposed by the CMap using a known list of seven drugs that have been previously tested in preclinical and/or clinical trials in mouse models and humans with DS, respectively. To test the specificity of the CMap data output for DS, we also checked the connectivity score of three additional drugs that are being used as treatments for other unrelated conditions, including riluzole (autism spectrum disorder), streptomycin (antibiotic)and metformin (type 2 diabetes).

## Results

### Gene Expression Analysis

#### Primary Effects in Tissues and Cell Types Derived from Humans with DS

The differentially-regulated genes at BH-FDR cut-offs of 5% and 20% are shown in the “Supplementary Results” section and “Supplementary Table 1”. A limited number of differentially-regulated genes were identified at an FDR of 5% (103, 8, 49 and 46 for the human amniocytes, Dp16, Ts65Dn and Ts1Cje mouse models, respectively). We therefore used an FDR threshold of 20%. This resulted in a higher number of differentially-regulated genes (786, 26, 74 and 71 for the human amniocytes, Dp16, Ts65Dn and Ts1Cje mouse models, respectively). We used these genes for the rest of the study to be able to identify commonly dysregulated genes between different human and mouse cell types and tissues. When we compared the expression of *Hsa*21 genes in multiple cell types and tissues derived from individuals with DS, we found that only a limited number of these genes were differentially-regulated in the cell types and tissues examined in this study (Supplementary Figure 1). All of these genes were located on the long arm of human chromosome 21 (21q) between *AP001347.6* and *DSTNP1*. Eight were non-protein coding genes. The largest number of differentially-expressed genes mapped to the 21q11-21q22.3 chromosomal region encompassing *LIPI* and *ZBTB21* (also known as *ZNF295*) and the remaining genes were distal to *ZBTB21* on 21q.3. Only two genes (*TTC3 and USP16*) were differentially-expressed in all human cell types and tissues, and 29 genes were overexpressed in at least two human cell types and/or tissues (Supplementary Figure 1).

#### Primary Effects in the Mouse Embryonic Forebrains

When the three mouse models were analyzed as a group, we found that a little more than a third of the *Hsa*21 orthologous genes on *Mmu*16 (i.e. 56 protein-coding and non-coding between *Lipi* and *Zbtb*21) were differentially-regulated during forebrain development. Eleven genes were consistently dysregulated in all three models, fifteen genes were common to the Dp16 and Ts65Dn models, and twenty-one were common between Ts1Cje and Ts65Dn. Each mouse model had uniquely dysregulated genes: four in Dp16, six in Ts1Cje and ten in Ts65Dn (Supplementary Table 2, Supplementary Figure 2).

#### Similarities and Differences Between Humans with DS and Mouse Models

**Figure 1:**
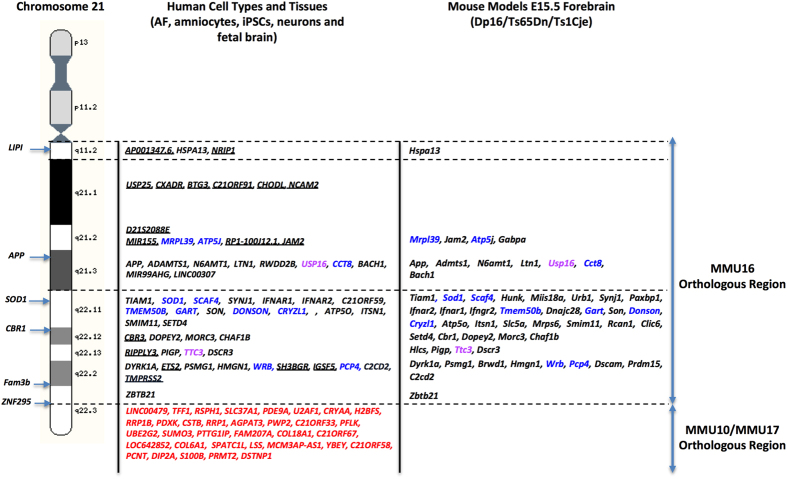
Primary Effects of Trisomy in Humans with DS and Mouse Models. Comparison of the Hsa21 and Mmu16 orthologous differentially regulated genes at FDR 20% in human cell types and tissues and embryonic forebrain from the Dp16, Ts65Dn and Ts1Cje mouse models of DS. Differentially regulated genes in all human and mouse cell types/tissues examined (Pink) and in at least two human cell types/tissues and two mouse models (Blue) are represented. It is important to notice that, in addition to the Hsa21 orthologous genes on the mouse Mmu16, a number of genes that are not triplicated in the Dp16, Ts65Dn and Ts1Cje mouse models and that are orthologous to the Mmu10 and Mmu17 in mouse are also significantly differentially regulated in humans with DS (Red).

Most differentially-expressed genes on *Hsa*21 were located between *LIPI* and *ZBTB21* in a region that is orthologous to the distal region of *Mmu16* that is duplicated to different extents in the Ts1Cje, Ts65Dn and Dp16 models (Fig. 1). Among these genes, only two (*USP16* and *TTC3*) were differentially-expressed in all human cell types and tissues, 11 were dysregulated in at least two human cell types/tissues and two mouse models and 18 genes were uniquely differentially- regulated in different human cell types and tissues (Fig. 1, Table 1).

Additionally, multiple genes on *Hsa*21 that are orthologous to mouse chromosomes 10 (*Mmu*10) and 17 (*Mmu*17) were also statistically significantly differentially-regulated in at least two human cell types and tissues. Three of these, *SLC37A1*, *PDE9A*, *RRP1B* are orthologous to *Mmu*17, and five are orthologous to *Mmu*10 (*PDXK*, *CSTB*, *YBEY*, *C21ORF33* and *UBE2G2)* (Fig. 1, Table 1).

#### Comparison of the Genome-Wide Effects of Trisomy in Human and Mouse Tissues

We found that human cell types and fetal brain showed important signature similarities particularly between iPSCs, iPSCs-derived neurons, cerebrum and cerebellum. Amniocytes from fetuses with DS showed some similarities with the fetal cerebrum and iPSCs, but to a lesser extent than iPSCs-derived neurons and the fetal cerebellum. Up-regulated gene lists showed more signature similarities than down-regulated genes (Table 2).

When the Ts1Cje, Ts65Dn and Dp16 mouse models were compared, we observed that embryonic forebrains showed molecular signature similarities. These similarities were more pronounced when up-regulated gene lists were compared between models.

When human cell types/tissues and mouse models transcriptome profiles were compared, we observed that signature similarities between species were less obvious compared to the similarities within each species. Up-regulated gene lists were more consistent between humans with DS and all mouse models.

**Figure 2:**
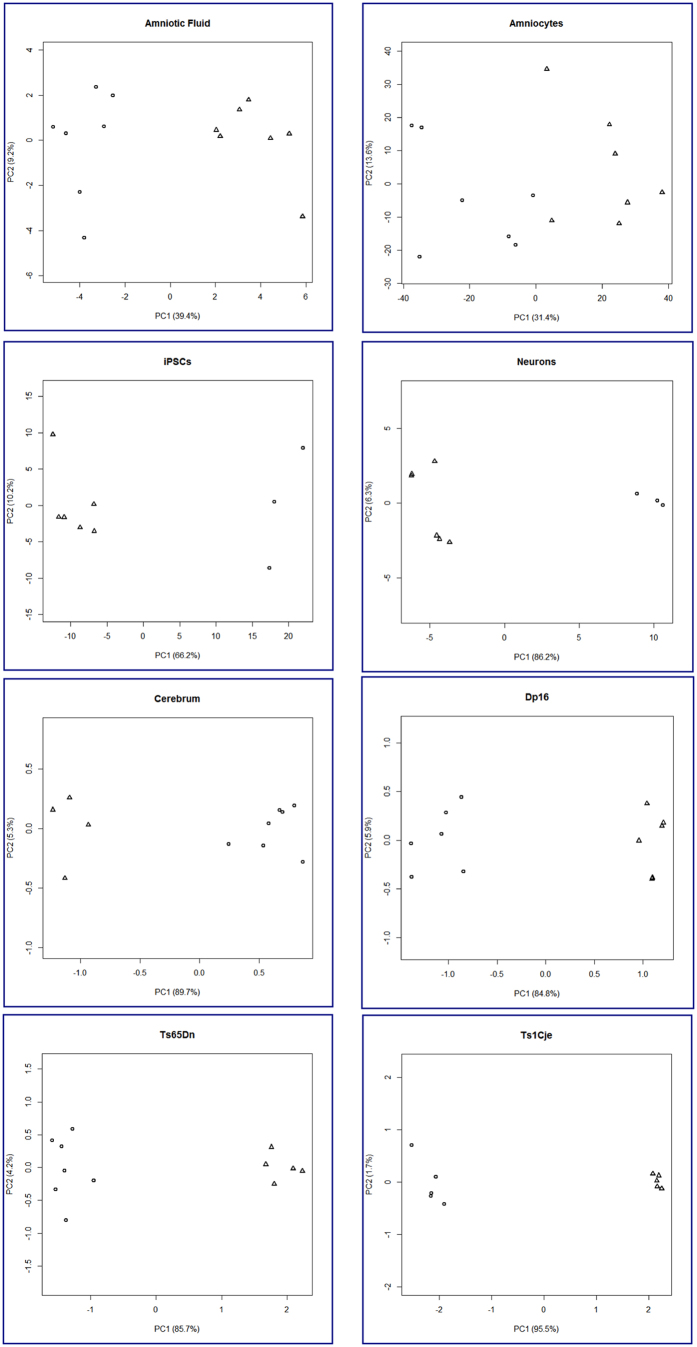
Principal Component Analysis (PCA) of cell types/tissues from humans with T21 and mouse models. PCA analysis of the differentially-regulated genes lists at FDR 20% shows a clear separation between euploid and trisomic samples in all tissues examined. Human cell types and tissues, particularly amniocytes from fetuses with T21 and sex and gestational age-matched euploid fetuses show more inter-individual variability than embryonic brains from the Dp16, Ts65Dn and Ts1Cje mouse models of DS.

We performed Principal Component Analysis (PCA) on the differentially regulated genes at FDR 20%, and observed that trisomic samples clustered together and were clearly separated from euploid samples in all cell types/tissues except amniocytes (Fig. 2).

### Pathway Analyses

#### Functional Pathway Abnormalities in Humans with DS

Using three different databases (DAVID, IPA and GSEA/DFLAT), we identified several key cellular processes and signaling pathways that are dysregulated in several cell types and tissues from humans with DS.

#### Abnormal Development in Human Cells and Tissues

In all examined datasets, gene expression changes highlighted general developmental delays affecting several organ systems, including the central nervous, skeletal, cardiovascular and reproductive systems. Major functional abnormalities were identified in the brain, as shown by the dysregulation of multiple genes (*TTC3*, *FOXG1*, *MBP*, *DLX1*, *SERPINF1*, among many others) (Table 2, Supplementary Tables 4, 5, 6). Neurogenesis and neuronal differentiation (axonal growth and synaptogenesis) were more significantly affected than myelination and gliogenesis in cell types and tissues examined (Table 2, Supplementary Tables 4, 5, 6).

#### Increased Transcriptional, Proteolytic and Proteasome Activity

Overexpression of a subset of *Hsa*21 genes led to genome-wide dysregulation of mRNA transcription through the activation of a large number of zinc finger genes (e.g., *ZNF248*, *ZNF37A*, *ZNF485* and *ZNF620*) and transcription factors belonging to the Forkhead family in all tissues examined (e.g., *FOXG1*, *FOXA1*, *FOXO3*, and *FOXO6*), the HOX family in iPSCs and iPSCs-derived neurons (*HOXC6*, *HOXB2* and *HOXB3*) and the SOX family (*SOX9*, *SOX11* and *SOX9*) in the fetal cerebellum and cerebrum. All tissues and cell types examined demonstrated a significant enrichment in genes associated with proteolysis and proteasome activity, including the ubiquitin-specific protease superfamily (e.g., *USP11*, *USP16*, *USP25*, *USP43*). Amniocytes from fetuses with DS showed abnormal expression of several genes encoding ubiquitin-protein ligases (e.g., *UBE2C, UBE2E1, UBE2L3*) that were not affected in iPSCs, iPSCs-derived neurons and fetal brain, except *UBE2G2*.

#### Cell Cycle and Cytoskeleton Organization Abnormalities

Amniocytes, iPSCs, and, to a lesser extent in IPSCs-derived neurons, were enriched in several genes implicated in the regulation of cell-cycle checkpoints during different phases particularly G1/S transition, including the gene encoding the mitotic checkpoint serine/threonine kinase BUB1 and several cyclin-dependent kinases (e.g., *CDKN3, CDK9, CDK12*) and cell division-cycle associated proteins (e.g., *CDCA5, CDCA3, CDC42*). Abnormal expression of CDK and CDC associated genes was not detected in post-mortem cerebrum and cerebellum from fetuses with DS. Cell cycle dysregulation was associated with abnormalities in cytoskeleton organization as shown by gene expression changes in several genes coding for spindle microtubules belonging to the kinesin superfamily (e.g., *KIF1B*, *KIF20A*, *KIF1A* and *KIF3A*) and a few genes that coded for dynein and myosin (e.g., *MYO7A*, *MYL9*, *DYNLRB1*, *DYNLRB2*).

#### Immune System Development and Inflammatory Response

Samples from fetuses with T21 demonstrated abnormal expression of several genes implicated in immune system development (e.g., *BRCA2*, *MLL*, *TAC1*, *IFNAR1*). Moreover, up-regulation of genes implicated in antigen processing and presentation (e.g., *HLA-DPB1* and *HLA-C* in the cerebellum and cerebrum; *HLA-DOA*, *HLA-A*, *HLA-B*, *HLA-F* and *HLA-J* in amniocytes) as well as inflammatory response (*SPP1* and *CXCL12* for the cerebrum and cerebellum; *IL10RB*, *TNFRSF10A* and *TNFAIP6* in iPSCs and iPSCs-derived neurons; *ILF3, IL1RAP, IL17RD, CXCL8* and *CRH* in amniocytes) was observed.

#### Mitochondrial Function, Anti-Oxidant Capacity and Apoptosis

Functional analysis highlighted a significant up-regulation of genes encoding for multiple mitochondrial proteins, including members of the NADH dehydrogenase family (*NDUFB6* and *NDUFC2* in amniocytes, *NDUFA5* in fetal cerebrum and cerebellum), 39S ribosomal family (e.g., *MRPL3*, *MRPL4*, *MRPL10* and *MRPL39*) and other genes known to play a key role in mitochondrial function (e.g., *ATP5O*, *ATP5J*).

Human cells and post-mortem brains showed significant up-regulation of several glutathione peroxidases (e.g., *GPX3*, *GPX5*, *GPX7*, *MGST2* and *GSTZ1*), oxido-reduction protein coding genes (e.g., *CYB5B*, *CYP4F11* and *CYP4Z1* in amniocytes; *COA7* and *CYP1B1* in iPSCs and iPSCs-derived neurons; *CYP4A11*, *CYP26A1*, *COX7A* and *UQCR11* in fetal brain), catalase (in iPSCs and neurons) and a number of genes implicated in the generation, metabolism and elimination of reactive oxygen species (e.g., *SOD1*, *TXNIP*, *PRDX5*, *TPO*, *PYROXD2*).

#### Activation of DNA Repair and Abnormal Kinetochore Organization Process

Amniocytes, iPSCs and iPSCs-derived neurons showed significant up-regulation of genes activated in response to DNA damage and that play a key role in DNA repair (e.g., *CHEK1*, *FANCD2*, *H2AFX* and *MPG* in amniocytes; *ATAD5*, *DDIT3*, *APC*, *SUPT16H* and *ALKBH3* in iPSCs and iPSCs-derived neurons). Additionally, an important number of genes implicated in kinetochore protein complex organization during mitotic spindle formation were highly enriched in amniocytes, iPSCs, iPSCs-derived neurons, and to a lesser extent in the fetal cerebrum and cerebellum, including the centromere protein family (e.g., *CENPA*, *CENPF*, *CENPO*, *CENPQ* and *CENPT*) and the kinesin protein family.

#### Dysregulation of Kinase/Phosphatase Balance and ATP-Binding

Consistent with the dysregulation of multiple cellular processes and signaling pathways, we observed a significant kinase/phosphatase imbalance in all affected human cells and tissues (Supplementary Table 7). The dysregulated kinases outnumbered phosphatases, however, we observed that kinases and phosphatases regulating the same signaling pathway were present in each cell type or tissue examined.

### Comparative Analysis of Cellular Processes and Pathway Abnormalities

Similarly to what we observed in human tissues, gene expression data from the embryonic day 15.5 forebrain of the Ts1Cje, Ts65Dn and Dp16 mouse models revealed that these models show several functional pathway similarities and differences when compared to each other, but also when compared to human cell types and fetal brains from fetuses with DS (Table 3).

#### Functional Similarities Between Human with DS and Mouse Models

**Figure 3:**
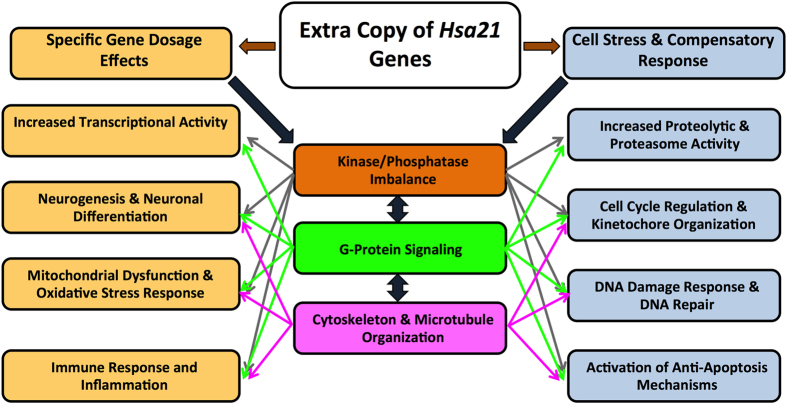
Functional Pathway Abnormalities in Humans with DS and Mouse Models. The presence of an extra copy of *Hsa*21 results in gene-dosage specific pathway changes concomitantly with a global cell stress response and the activation of compensatory mechanisms. There is no significance to the colors used in this figure.

Even though the differentially-regulated genes were not fully conserved among the cell types, tissues and species examined, we were able to identify several cellular processes and pathway abnormalities that were common to both humans with DS and mouse models. A global dysregulation of transcriptional and proteasome activity was consistently observed, along with kinase/phosphatase imbalances, immune system activation, mitochondrial dysfunction and the activation of anti-oxidant defenses and anti-apoptotic mechanisms in all tissues examined (Table 3, Fig. 3). Additionally, abnormal expression of gene clusters associated with abnormal central nervous system development (particularly axogenesis and synaptogenesis), G-protein signaling (i.e. represented by olfactory receptors in mouse models that are not conserved in human tissues) and synaptic plasticity were observed in both humans with DS and mouse models (Table 3, Fig. 3).

#### Functional Abnormalities in a Specific Human Cell Type/Tissue or Mouse Model

Comparative pathway analyses highlighted specific signaling pathways that are perturbed in specific human cell types/tissues or mouse models (Table 3). Genes that play a key role in cell cycle checkpoint regulation (e.g., *CDKN3*, *CDK9*, *CDK12*, *CDCA5*, *CDCA3*, *CDC42*), kinetochore organization (e.g., *KIF1B*, *KIF20A*, *KIF1A* and *KIF3A*) and DNA repair (e.g., *CHEK1*, *CHAF1B*, *FANCD2*, *ESCO2*, *NEIL3*, *APC* and *ALKBH3*) were highly enriched in the human amniocytes, iPSCs, iPSCs-derived neurons and in the Ts1Cje mouse model. However, they were not affected in the human fetal cerebrum, cerebellum or the Ts65Dn and Dp16 mouse models.

Genes involved in the regulation of neurogenesis (e.g., *KIT*, *DLX1*, *DLX2*, *ID4*, *NEUROD1*, *BARHL2* and *IRX3*) were more significantly dysregulated in iPSCs-derived neurons and fetal brains derived from individuals with DS as well as in the Ts65Dn mouse model, but they were not significantly affected in the Ts1Cje and Dp16 mouse models. Amine transmembrane transport, however, was perturbed in human tissues and in the Ts1Cje embryonic brain.

Finally, regulation of cellular homeostatic processes (most importantly, calcium ion homeostasis) was significantly affected in all human DS cell types and tissues examined in addition to the Dp16 mouse model.

### Small Molecule Screening

#### Molecules Predicted to Rescue Abnormal Signatures in Humans with DS

**Figure 4:**
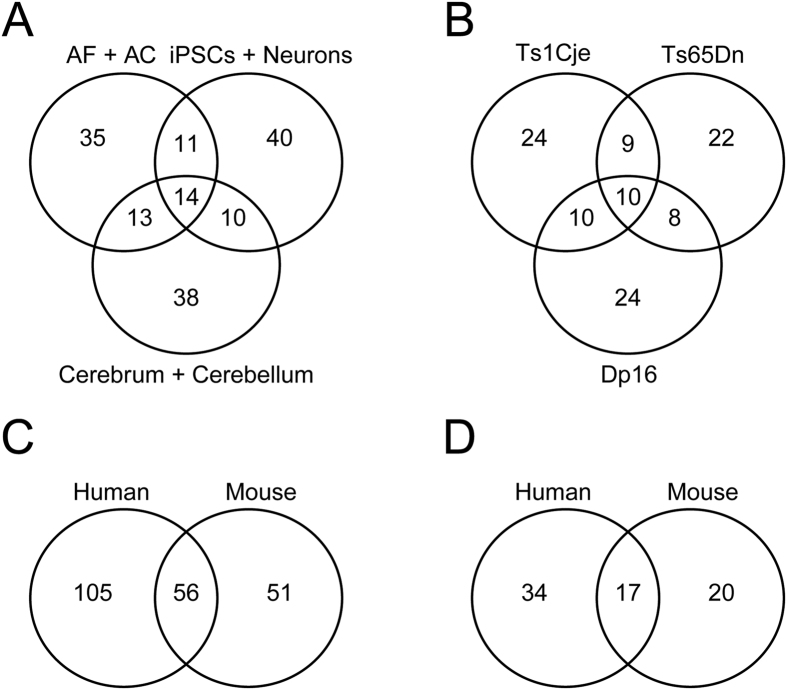
Molecules Predicted to Treat Dysregulated Pathways in Different Cell Types and Brain Tissue From Humans With DS and Mouse Models. Venn diagrams representing the number of small molecules predicted by the CMap to reverse abnormal transcriptome signatures in at least one human cell type and post-mortem fetal brain (**A**) and at least one mouse model embryonic day 15.5 forebrain (**B**). Note that the total number of drugs identified is 161 for humans and 107 for mice. Common molecules predicted to reverse the transcriptome signature in at least one human cell type or tissue and mouse model are represented in (**C**). Out of the 161 drugs identified in humans and 107 in mouse models, 56 molecules were common to humans with DS and mouse models. Small molecules predicted to reverse the transcriptome signature in at least two human and mouse model cell types/tissues are represented in (**D**). Out of 56 drugs common in humans with DS and mouse models, 17 were predicted to rescue the transcriptome signature in at least two human cell types and tissues and two mouse models.

Using a cut-off value of −0.7, the CMap predicted that on average, 41 molecules could potentially improve gene expression and pathway abnormalities in human cell types or fetal brain (38 for AF, 46 for amniocytes, 45 for iPSCs, 40 for iPSCs-derived neurons, 28 for cerebellum and 50 for cerebrum). When two human cell types and/or tissues were compared, on average nine drugs were predicted to reverse the transcriptome in the two tissues analyzed. This overlap is significantly higher than the 1.3 that would be expected for two random independent comparisons (p-value < 0.001, Fisher test) (Fig. 4A).

#### Molecules Predicted to Rescue Abnormal Signatures in Mouse Models

Using the same cut-off value, the CMap predicted that on average 51 molecules could potentially improve gene expression and pathways in the mouse models (53, 49 and 52 for Ts1Cje, Ts65Dn and Dp16, respectively). When two mouse models were compared, on average, 19 drugs were predicted to reverse the transcriptome in the two models analyzed (overlap significant with a p-value < 0.001, Fisher test). Ten drugs were highly enriched in the three mouse models at a connectivity score of <−0.7 (Fig. 4B).

#### Molecules Predicted to Rescue Abnormal Signatures in Humans and Mice

We compared the lists of molecules with high negative connectivity scores (CS < −0.7) in all cell types and tissues examined in this study. Fifty-six molecules were predicted to reverse the abnormal transcriptome signature in at least one human cell type or tissue and one mouse model, including apigenin (Fig. 4C). When we applied stricter criteria (two human cell types or tissues and two mouse models), we identified seventeen drugs with negative connectivity scores (Fig. 4D).

#### Connectivity Map Enrichment Scores for Molecules Tested Previously

CMap analysis of seven drugs previously used in preclinical and clinical trials to improve cognition in DS revealed that antioxidants like vitamins A (average CS = −0.395 in mice and −0.177 in humans) and C (average CS = −0.490 in mice and −0.078 in humans), minocycline (average CS = −0.421 and −0.087 in mice and humans, respectively) and MK-801 (average CS = −0.592 in mice and −0.151 in humans) had higher negative connectivity scores in the mouse models than in human tissues and would be predicted to have only a mild therapeutic effect on the pathway abnormalities in humans with DS. Folinic acid (average CS = 0.231 and 0.402 in mice and humans, respectively) and fluoxetine (average CS = 0.447 and 0.438 in mice and humans, respectively) showed positive enrichment scores in most human and mouse cell types and tissues examined (Table 4). *This implies that treatment with either of these molecules would exaggerate the phenotype of DS*. The opposite, however, was true for memantine, an NMDAR antagonist that showed a higher connectivity score in human cell types and tissues (average CS = −0.33) particularly in iPSCs, iPSCs-derived neurons and fetal cerebellum when compared to mouse embryonic forebrain (average CS = 0.116). Finally, piracetam (a derivative of GABA) and apigenin (a flavone) had negative connectivity scores in 8 and 7 cell types and tissues, respectively (average CS = −0.417 for apigenin and −0.178), with apigenin having more average negative connectivity scores in humans and mouse models (−0.296 and −0.692, respectively) than piracetam (−0.216 and −0.406, respectively). Apigenin had the best negative connectivity scores in most tissues, except in human AF and cerebellum.

As a negative control, we analyzed enrichment scores for three randomly selected drugs (riluzole, streptomycinand metformin) that are currently in preclinical and clinical use for conditions other than DS (Table 4). They would be predicted to have a less therapeutic effect (manifested as positive connectivity scores) than the other molecules selected using gene expression data and the CMap to treat DS. As expected, these three molecules displayed positive average connectivity scores in humans (average CS = 0.195, 0.375 and 0.145, respectively) and mouse models (average CS = 0.378, 0.470 and 0.254, respectively).

Table 4 details the connectivity scores for each drug, cell type and tissue and can be used to find cell type or region-specific efficacious drugs. The connectivity scores can be used to develop hypotheses regarding the effects of a particular treatment *in vitro* and *in vivo* models before moving forward to a human clinical trial.

## Discussion

In this study, we used a novel integrated human/murine systems biology approach to interrogate nine different gene expression data sets and identify commonly dysregulated candidate signaling pathways and cellular processes that can be targeted for prenatal therapeutic interventions in DS. Our results highlighted the importance of *USP16* and *TTC3* as consistently dysregulated genes in all cells and tissues analyzed. We identified multiple dysregulated pathways that can serve as therapeutic targets. We successfully used the human CMap database and created an adapted mouse version to identify FDA-approved drugs that can specifically target these pathways and be tested in pre-clinical trials.

### Primary Effects of Trisomy in Human with DS and Mouse Model

#### Limited Number of Hsa21 and Mmu16 Orthologous Genes are Overexpressed

We first compared the expression profiles of *Hsa*21 genes in amniocytes derived from second trimester fetuses to other cell types and tissues, including AF supernatant, iPSCs, iPSCs-derived neurons, post-mortem cerebrum and cerebellum from humans with DS. We found that not all *Hsa*21 genes were overexpressed in a gene dosage-dependent manner (i.e., a 1.5 fold increase). A few reports in the literature have described comparable effects in fetal hearts, fibroblasts, lymphoblastoid cell lines and adult post-mortem brains from humans with DS^18^,^19^,^20^. In their meta-analysis of thirty different gene expression studies of different DS model systems (human cell lines, human tissues and mouse models), Vilardell *et al*. identified only 77 *Hsa*21 genes that were significantly differentially-regulated using their numerical scoring methods^21^.

Similar to what has been observed in humans with DS, duplication of the *Hsa*21 orthologous genes on mouse *Mmu*16 did not result in the overexpression of all of the duplicated genes in the embryonic brain in several mouse models, including Ts1Cje, Ts65Dn and Dp16. Only 33 *Mmu*16 genes in Ts1Cje (out of 77 trisomic genes), 46 genes in Ts65Dn (out of 128 trisomic genes) and 19 in Dp16 (out of 145 trisomic genes) were differentially-regulated^12^,^22^. Our studies have been corroborated by other groups that reported up-regulation of only a subset of *Mmu*16 triplicated genes in the developing Ts1Cje cerebellum and tissues from Ts65Dn, including brain and heart^23^,^24^,^25^,^26^,^27^.

#### Hsa21 Orthologous Genes on Mmu10 and Mmu17 are Overexpressed Only in Humans with DS

Human cell types and tissues also displayed up-regulation of several genes that are orthologous to *Mmu*10 (e.g., *PDXK*, *CSTB*, *YBEY* and *UBE2G2*) and *Mmu*17 (e.g., *SLC3A7*, *PDE9A* and *RRP1B*). Most of these genes were differentially-regulated in other cell types such as fibroblasts and lymphoblastoid cell lines, fetal heart and adult brain, suggesting their importance in DS pathophysiology^17^,^18^,^19^,^20^.

In mice, two models carrying the duplication of HSA21 genes orthologous to genes on the mouse MMU17 (Dp17 and Ts1Yah) showed altered hippocampal long-term potentiation (LTP) and some neurocognitive deficits suggesting the importance of this region in the DS-associated phenotype^28^,^29^,^30^. However, trisomy of the MMU10 orthologous region did not yield hippocampal-dependent cognitive deficits in the Dp10 mouse model of DS, but induced an abnormal protein expression profile in the cerebellum warranting potential implication of this region in the motor coordination deficits associated with DS^29^,^30^,^31^.

#### Candidate Genes for Fetal Development in Humans with DS

To identify candidate genes that affect fetal development in DS, we found only two genes (*USP16* and *TTC3*) were differentially-regulated in all human cell types and tissues examined. This suggests that they are important in the phenotype of DS. *TTC3* is ubiquitously expressed during embryonic development in the mouse, particularly in the central nervous system^32^. *TTC3* plays a key role in the regulation of neuronal differentiation and its overexpression prevents neurite outgrowth in differentiating primary neurons^33^. TTC3 protein mediates the ubiquitination and subsequent degradation of phosphorylated Akt (AKT1, AKT2 and AKT3) in the nucleus^34^. Additionally, emerging experimental evidence suggests that the USP16 protein interacts with the E3 ubiquitin protein ligase HERC to regulate histone 2A deubiquitination during DNA-repair to regulate and terminate the repair signal^35^. USP16 regulates kinetochore localization of PLK1 to promote proper chromosome alignment during mitosis, and its knock-out in Hela cells resulted in slow cell growth and defects in the mitotic phase of the cell cycle^36^,^37^. USP16 knock-out mice die *in utero* as early as embryonic day 6 (https://www.jax.org/strain/017374). *USP16* overexpression reduced self-renewal in human and Ts65Dn fibroblasts and primary neuroprogenitors and resulted in accelerated senescence in these cells^38^,^39^. In addition to *USP16* and *TTC3*, eleven genes were differentially-regulated in at least two human tissues and two mouse models, suggesting that phenotypic changes in fetuses with DS are not triggered by the overexpression of a single gene but rather by the cumulative effects of multiple genes on *Hsa*21.

### Functional Pathway Abnormalities in Humans with DS and Mouse Models

#### Gene Dosage-Specific Effects and the Activation of Global Cell Stress and Compensatory Mechanisms in DS

Our functional pathway analyses of multiple human cell types and tissues indicated that the presence of an extra copy of *Hsa*21 resulted in very complex downstream effects that can be generalized into two different categories: (1) increases in the transcriptional activity of *Hsa*21 and target genes, leading to abnormal neurogenesis and neuronal differentiation, mitochondrial dysfunction and oxidative stress response and increased inflammation; and (2) global cellular stress response with the activation of compensatory mechanisms, including DNA-repair signaling, abnormal regulation of cell cycle checkpoints and kinetochore organization, increased proteolytic activity and the activation of anti-apoptotic genes. The dichotomy between these two categories is not simple to explain as they are activated simultaneously and triggered by an imbalance of the kinase/phosphatase homeostasis, G-protein signaling abnormalities and the disruption of the cytoskeleton and microtubule networks.

In their meta-analysis of transcriptomic datasets obtained from trisomies in different species, including fission yeast, plants (*Arabidopsis thaliana*), mouse embryonic fibroblasts (trisomies of *Mmu*1, 13 16 and 19), and humans (trisomies of *Hsa*13, 18 and 21), Sheltzer *et al*. identified striking similarities among conserved stress response mechanisms and cell cycle abnormalities^40^. In humans, trisomies of different autosomes, including 21 (Down syndrome), 13 (Patau syndrome) and 18 (Edwards syndrome) result in some phenotypic similarities (postnatal growth failure, cardiac structural abnormalities and intellectual disability) and differences (different specific cognitive abnormalities, dysmorphic facial features and associated comorbidities)^41^,^42^. These similarities and differences could be the result of both gene-dosage specific effects and global stress response and compensatory mechanisms that can provide important insights for future therapeutic interventions.

#### Pathway Similarities and Differences in Humans with DS and Mouse Models

Trisomy of *Hsa*21 in humans and orthologous genes in mice resulted in pathway similarities and differences between the two species, but also between different cell types within the same species (Table 3). Cell cycle, kinetochore organization and DNA repair were significantly affected in human amniocytes, iPSCs and the Ts1Cje mouse model; however, neurogenesis and neuronal differentiation were more affected in human iPSCs-derived neurons and fetal brains as well as the Ts65Dn mouse model.

In agreement with our gene expression studies, cellular similarities and differences were previously reported between humans with DS and mouse models. Neurogenesis, synaptogenesis and axonal growth are more severely affected in human fetuses with DS and the Ts65Dn mouse model, however, milder neurogenesis defects were reported in the Ts1Cje model and no defects were observed in the Dp16 mouse model^43^,^44^,^45^,^46^,^47^,^48^,^49^. Additionally, iPSCs and neurons derived from monozygotic discordant twins shed light on several phenotypic abnormalities in DS stem cells, including cell proliferation delay, excitatory/inhibitory transmission imbalance and higher sensitivity to oxidative stress induced apoptosis, some of which were impossible to assess directly in the brain of humans with DS^7^,^50^,^51^. Similar to the iPSCs, amniocytes from fetuses with DS display cell proliferation defects, early senescence and shorter telomeres compared to euploid fetuses^52^,^53^,^54^.

Because of these similarities and differences between human and murine cell types and tissues, our data and the literature suggest that an integrated approach using material from both species as sources of RNA is crucial to better understand the pathophysiology of DS and develop effective treatments. In this context, iPSCs from human with DS have been used to evaluate the corrective effects of XIST mediated chromosomal silencing or DYRK1A kinase inhibition using EGCG^50^,^51^.

### Molecular Screening Using the Connectivity Map

Molecular screening using the human and mouse adapted CMaps identified seventeen drugs that are predicted to reverse signature and pathway abnormalities in both humans with DS and in mouse models. To the best of our knowledge, this work represents the only application of prenatal treatment hypothesis testing using the CMap. Other groups, however, have used the CMap to repurpose drugs for neurodegenerative diseases, including Alzheimer disease and Parkinson disease^10^,^55^,^56^.

During the last ten years, there has been increasing interest in developing therapeutic interventions to improve neurocognition in individuals with DS^2^. Multiple drugs have been successfully used in the Ts65Dn mouse models, however, they lacked efficacy in human clinical trials^2^. A close examination of the CMap connectivity scores for these drugs provides some insights as to why these molecules did not have a measurable therapeutic effect. Some of these drugs were not predicted to rescue the molecular signature in humans with DS and mouse models (vitamins and folinic acid) and others were predicted to rescue the transcriptome signature in mouse models but not in humans with DS (minocycline, MK-801). This might provide an explanation as to why certain drugs resulted in clinical improvement in the Ts65Dn mouse models, but failed to positively affect neurocognition in humans with DS^56^,^57^,^58^,^59^.

Piracetam gave negative connectivity scores in eight of the nine examined tissues with average scores of −0.216 and −0.406 in humans with DS and mouse models. Moran *et al*.^60^ tested the effects of piracetam in the Ts65Dn mouse model. These results were mixed: piracetam treatment improved the performance of Ts65Dn mice in the visible platform version of the Morris water maze test and blocked the beginning of the dark phase hyperactivity seen in these mice. In contrast, piracetam did not improve the performance of Ts65Dn mice in the hidden platform version of the MWM. Additionally, piracetam treatment started at 4 weeks of age and mice were tested around 10 weeks of age. There is no information available on the effects of piracetam on Ts1Cje and Dp16 mice. Furthermore, the effects of piracetam treatment during the fetal and neonatal periods of life have not yet been investigated. More detailed behavioral and molecular studies on multiple mouse models are needed to better investigate the effects of piracetam. With regards to humans, only one double-blinded human clinical trial was conducted in a cohort of 25 children with DS of which only 18 completed the study^61^. The authors analyzed 72 primary outcome measures of attention, memory, perceptual abilities, executive function and fine motor skills. Forty-six of these measures produced better results with the piracetam group compared to the placebo group. Additionally, 11 of the 18 parents reported cognitive improvement with piracetam and only two parents reported an improvement with placebo^62^,^63^.

Surprisingly, fluoxetine gave positive enrichment scores in all tissues examined and the most positive average connectivity score (0.441). Previous studies reported that prenatal treatment with fluoxetine had long lasting therapeutic effects in the Ts65Dn mouse model^64^,^65^. Another study on adult Ts65Dn mice, however, revealed that fluoxetine treatment at 0.2 mg/ml did not improve behavioral deficits, and resulted in seizures (in three Ts65Dn mice) and death (in four Ts65Dn animals), respectively^66^. Fluoxetine has not been evaluated in other mouse models of DS. There are no human studies of fluoxetine for DS in the literature. A clinical trial of prenatal treatment with fluoxetine is just starting to enroll subjects in the United States. Our CMap results would suggest that this treatment may not be helpful and could be potentially harmful.

N-methyl-D-aspartate (NMDA) receptor antagonists MK801 and memantine were previously tested as potential treatments of adult phenotypic deficits in the Ts65Dn mouse model of Down syndrome. Both treatments induced a significant increase of locomoter activity in Ts65Dn adult mice^67^. Additionally, memantine treatment improved Ts65Dn performances in the Morris water maze test and rescued the number of vGlut1 granule cells in the hippocampus of aged Ts65Dn mice^68^. MK-801 and memantine have not been evaluated in other mouse models of DS, and their safety and efficacy for prenatal treatment is still unknown. Two randomized double-blinded studies tested the effect of 16-weeks memantine treatment on the cognitive and adaptive functions of adults with Down syndrome^69^,^70^. Although no significant differences were observed in the primary outcome measures, the authors reported significant improvement in one of the secondary measures (California Verbal Learning Test-II) after treatment.

As a proof of principle for our integrated approach, we tested the effects of apigenin, one of the molecules predicted by the CMap to treat dysregulated pathways in DS, on human amniocytes derived from fetuses with DS and on the Ts1Cje mouse model of DS. Apigenin treatment reduced oxidative stress in DS amniocytes and improved some aspects of brain morphogenesis, gene expression and postnatal behavior in the Ts1Cje mouse model (manuscript in preparation).

At the present stage of knowledge, the connectivity and pathways maps are only a part of an unknown global network dysregulation; therefore, discrepancies between our results and some published studies might be linked to unknown connections. Additionally, analyzing the transcriptome modifications gives only one picture of the problem as translational modifications can create protein variations in ways different from RNA variations, and as protein networks may react differently to drugs than RNAs due to protein modifiers acting upon protein stability or protein interactants.

## Conclusions

In this study, we used a novel human/mouse integrated approach to demonstrate both similarities and differences in gene expression and signaling pathways in humans with DS and mouse models. These similarities and differences should be taken into account when designing and testing therapeutic interventions with drugs that can specifically target these pathway abnormalities. Here, we used the human CMap to objectively discover and repurpose safe compounds to test hypotheses regarding prenatal treatment of DS. The CMap search identified 17 high priority molecules predicted to reverse pathway changes in both human cells and mouse models. To determine the potential beneficial and harmful effects of these compounds, we are currently testing their safety and efficacy *in vitro* (amniocytes and iPSCs from fetuses with DS) and *in vivo* (in multiple mouse models of DS).

## Additional Information

**How to cite this article**: Guedj, F. *et al*. An Integrated Human/Murine Transcriptome and Pathway Approach To Identify Prenatal Treatments For Down Syndrome. *Sci. Rep*. **6**, 32353; doi: 10.1038/srep32353 (2016).

## Supplementary Material

Supplementary Information

Supplementary Table S1

Supplementary Table S2

Supplementary Table S3

Supplementary Table S4

Supplementary Table S5

Supplementary Table S6

Supplementary Table S7

## Figures and Tables

**Table 1:** Similarities and Differences in Differentially Regulated Genes in Humans With DS and Mouse Models.

Cell Types or Tissues Analyzed	Dp16 E15.5 Forebrain	Ts65Dn E15.5 Forebrain	Ts1Cje E15.5 Forebrain	*Hsa*21 Orthologs on MMU10 & MMU17
Human Amniotic Fluid & Amniocytes	*HSPA13, **MRPL39**, SON, **DONSON**, **CRYZL1**, **TTC3**, DYRK1A, ZBTB21*	*ATP5J, APP, LTN1, USP16, CCT8, SOD1, **SCAF4**, SYNJ1, IFNAR2, IFNAR1, **GART**, SON, **DONSON**, **CRYZL1**, CHAF1B, **TTC3**, DSCR3, DYRK1A, HMGN1, **WRB**, ZBTB21*	***SCAF4***, *SYNJ1, IFNAR2, IFNAR1, **GART***, *SON, **DONSON**, **CRYZL1***, ***TTC3***, *DSCR3, DYRK1A, **WRB***, *ZBTB21*	***RRP1B**, **PDXK**, **YBEY**, **C21ORF33**, **UBE2G2***
Human iPSCs & Neurons	*HSPA13, **DONSON**, **CRYZL1**, **TTC3**, PSMG1, PCP4*	***MRPL39**, ATP5J, SOD1, **TMEM50B**, **DONSON**, **CRYZL1**, ATP5O, DOPEY2, MORC3, **TTC3**, PSMG1, **WRB***	***TMEM50B**, **DONSON**, **CRYZL1**, ATP5O, DOPEY2, MORC3, PIGP, **TTC3**, PSMG1, **WRB***	***SLC37A1**, **PDE9A**, **PDXK**, **CSTB***
Human Fetal Cerebrum & Cerebellum	***MRPL39**, JAM2, N6AMT1, **TTC3**, PCP4*	***MRPL39**, USP16, JAM2, ADAMTS1, RIPPLY3, **TTC3***	***SCAF4**, **TMEM50B**, **GART**, ITSN1, SMIM11, SETD4, **TTC3***	***SLC37A**, **PDE9A**, **CSTB**, **RRP1B**, **C21ORF33**, **YBEY**, **UBE2G2***

Differentially regulated genes in both human tissues paired in each row and each mouse model are represented in bold. For Hsa21 genes not orthologous to the mouse Mmu16 that differentially regulated in human cell types/tissues are represented in bold (Mmu10 orthologous genes) and bold underlined (Mmu10 orthologous genes).

**Table 2:**
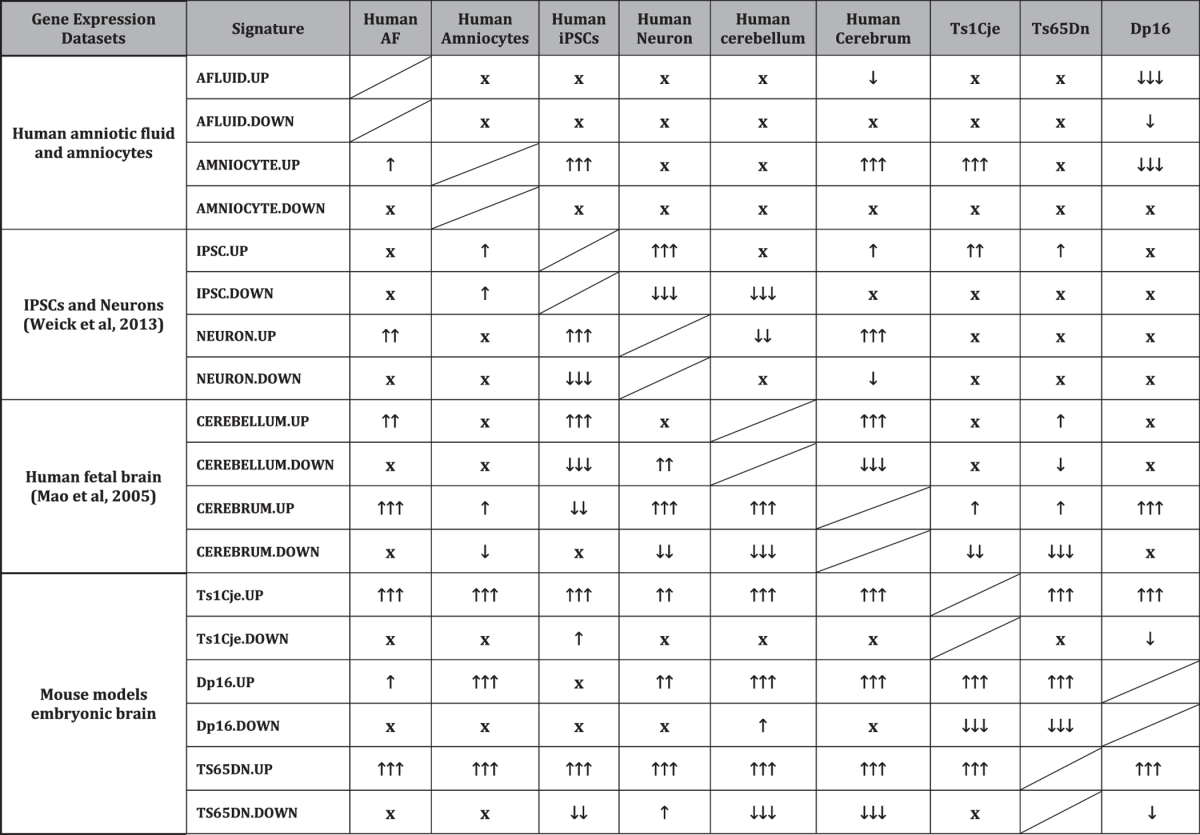
Comparison of the Genome-Wide Effects of Trisomy in Humans With DS and Mouse Models.

The genome-wide effects of trisomy were analyzed using the Gene Set Enrichment Analysis (GSEA) database. Up-regulated gene sets are indicated in red, down-regulated gene sets indicated in green and unchanged gene sets are represented as (X). The extent of up-regulation and down-regulation is indicated with arrows (one arrow for p < 0.05, two arrows for p < 0.01 and three arrows for p < 0.001).

**Table 3:** Cellular Processes and Functional Pathway Similarities and Differences in Humans With DS and Mouse Models.

Cellular Process/Pathway	DS AF & Amniocytes	DS iPSCs	DS Neurons	DS Cerebellum	DS Cerebrum	Ts1Cje E15.5 Brain	Ts65Dn E15.5 Brain	Dp16 E15.5 brain
Transcriptional Activity	*	**	***	*	*	*	***	*
Neurogenesis	*	*	**	***	**	—	**	—
Neuronal Differentiation	*	*	***	***	***	*	***	*
Mitochondrial Function	*	*	*	*	*	*	*	**
Response to Oxidative Stress	*	***	*	**	**	**	*	**
Immune Response/Inflammation	**	***	*	—	*	*	*	*
Proteolysis & Proteasome Activity	**	**	*	—	—	—	**	*
Cell Cycle Regulation & Kinetochore Organization	***	*	—	—	—	***	*	*
Regulation of Apoptosis	**	*	*	*	*	*	*	*
Kinase & Phosphatase Activity	**	*	*	*	*	***	*	*
G-Protein Signaling	*	*	*	*	*	*	*	*
Cytoskeleton Organization	***	**	*	**	*	***	*	*
Amine Transmembrane Transport	—	*	*	*	*	**	—	—
Cellular Homeostasis/Calcium Ion Homeostasis	*	**	*	**	**	—	—	*

Pathway similarities and differences between human cell types/tissues and mouse models are indicated as follows: **(−)**: No Enrichment; (*): Enrichment score between 0.5 and 1; (**): Enrichment score between 1 and 2; (***): Enrichment score bigger than 2. Enrichment score significance was defined using the binomial test for DAVID, the Kolmogorov–Smirnov test for GSEA, and Fisher test for IPA.

**Table 4:** Connectivity Map Enrichment Scores for Drugs used in preclinical and clinical trials in the literature.

Drug Name (Category)	DS AF	DS Amniocytes	DS iPSCs	DS Neurons	DS Cerebellum	DS Cerebrum	Ts1Cje E15.5 Brain	Ts65Dn E15.5 Brain	Dp16 E15.5 brain	Average Score
Apigenin (Flavone)	0.50	−0.862	−0.671	−0.536	0.576	−0.782	−0.659	−0.748	−0.668	**−0.417**
MK-801 (NMDAR agonist)	−0.265	−0.454	0.298	−0.311	0.270	−0.446	−0.567	−0.580	−0.63	**−0.298**
Vitamins A (Antioxidant)	0.150	−0.267	−0.251	−0.190	−0.174	−0.327	−0.412	−0.284	−0.488	**−0.249**
Vitamins C (Antioxidant)	−0.267	0.575	0.526	−0.384	−0.339	−0.579	−0.585	−0.444	−0.441	**−0.215**
Minocycline (Anti-inflammatory)	−0.327	0.324	0.368	−0.282	−0.327	−0.279	−0.505	−0.397	−0.362	**−0.199**
Piracetam (Derivative of GABA)	0.378	−0.289	−0.392	−0.431	−0.224	−0.29	−0.367	−0.575	−0.276	**−0.178**
Memantine (NMDAR antagonist)	0.354	0.288	−0.350	−0.523	−0.476	0.508	0.281	0.287	−0.219	**0.017**
Vitamins E (Antioxidant)	0.349	0.363	0.396	−0.220	−0.442	−0.241	0.298	0.297	0.292	**0.121**
Metformin (Type 2 Diabetes)	0.351	0.385	0.355	0.215	−0.222	−0.217	0.290	0.286	0.187	**0.181**
Riluzole (Autism spectrum disorder)	0.258	−0.251	0.175	0.382	0.258	0.354	0.319	0.549	0.266	**0.256**
Folinic Acid	0.476	0.743	0.747	0.752	−0.572	0.266	0.545	0.352	−0.205	**0.345**
Streptomycin (Antibiotic)	0.509	0.492	−0.253	0.647	0.283	0.570	0.366	0.423	0.621	**0.406**
Fluoxetine (Serotonin reuptake inhibitor)	0.436	0.370	0.424	0.747	0.209	0.444	0.482	0.433	0.426	**0.441**

A negative connectivity score suggests that a particular drug reverses the transcriptomic signature in samples derived from humans with DS and mouse models. A complete rescue is associated with a connectivity score of −1.00. A positive connectivity score suggests that a particular drug will exaggerate the gene expression abnormalities in DS. A null score suggests no effects on the DS signature. CMap drug list was ranked from the highest to the lowest average connectivity score for all the cell types and tissues examined.
