# Supplementary material for: An Integrated Human/Murine Transcriptome and Pathway Approach To Identify Prenatal Treatments For Down Syndrome
Source: Sci Rep. 2016 Sep 2;6:32353. doi: 10.1038/srep32353 (PMC5009456; doi:10.1038/srep32353)
Supplement: Supplementary Information [file srep32353-s1.pdf]

An Integrated Human/Murine Transcriptome and Pathway Approach To Identify Prenatal Treatments For Down Syndrome

Faycal Guedj[1*], Jeroen LA Pennings[2], Lauren J Massingham[1], Heather C Wick[3], Ashley E Siegel[1], Umadevi Tantravahi[4], Diana W Bianchi[1]**

Supplementary Results

Second Trimester Amniocytes Information

Pair	Sample ID	Gestational Age	Karyotype
**Pair 1**	**JA-186**	18 + 1/7	46XX, 2N
	**AR-140**	18 + 3/7	47XX, T21
**Pair 2**	**KS-188**	16 + 3/7	46XX, 2N
	**PH-182**	16 + 4/7	47XX, T21
**Pair 3**	**MK-213**	15 + 3/7	46XX, 2N
	**KF-121**	15 + 2/7	47XX, T21
**Pair 4**	**NM-344**	20 + 5/7	46XX, 2N
	**FF-337**	21	47XX, T21
**Pair 5**	**NS-335**	18 + 5/7	46XY, 2N
	**CQ-908**	19 + 2/7	47XY, T21
**Pair 6**	**AW-372**	18 + 1/7	46XY, 2N
	**AF09-122**	19 + 1/7	47XY, T21
**Pair 7**	**CO-384**	19	46XY, 2N
	**DC-317**	19 + 2/7	47XY, T21

Human amniocytes from human fetuses with DS show significant genome-wide expression dysregulation

We used the Human Genome U133 2.0 Plus arrays to compare gene expression changes in second trimester human amniocytes derived from fetuses diagnosed with trisomy 21 (N=7) and age and sex-matched euploid fetuses (N=7).

Limited primary effect of trisomy in amniocytes derived from fetuses with DS

Human chromosome 21 (HSA21) is the smallest autosome with 233 coding and 446 non-coding genes (http://www.ensembl.org/index.html). We found that only 47 genes (44 up-regulated and 3 down-regulated) and one microRNA (*MIR99AHG*) mapping to HAS21 were statistically significantly differentially regulated in amniocytes from fetuses with DS compared to euploid gestational age and sex-matched controls. All these genes were located on the long arm (21q) between *AP001347.6* and *DSTNP1*, among which 40 were protein coding and 7 non-protein coding genes. Two genes (*AP001347.6* and *HSPA13*) were located on 21q11, 10 genes and *MIR99AHG* on 21q21 and the remaining 35 on 21q22 between *TIAM1* and *DSTNP1* (chromosomal position 3,1118,416 to 4,6654,022) corresponding to the 15.5 Kb most distal part of HSA21 (Figure1, Table1). The average mean expression of all the HSA21 up-regulated genes was 2.47, a ratio that is higher than the expected gene dosage effect (i.e. 1.50) with different expression levels ranging from 1.06 (for *HMGN1*) to 5.65 (for *AGPAT3*) (Table1).

Significant genome-wide secondary effect in amniocytes derived from fetuses with DS

Despite the limited number of differentially regulated HSA21 genes, we observed an important genome-wide effect of the trisomy on other chromosomes with 1,105 genes (735 up-regulated and 370 down-regulated) statistically significantly differentially regulated in human amniocytes from fetuses with DS compared to euploid fetuses at the BH-FDR of 20% (Supplementary Table 1). This genome-wide secondary effect was detected on all the chromosomes of the genome with varying numbers of differentially regulated genes ranging from 23 genes (12 up-regulated and 11 down-regulated) for chromosome 18 to 112 genes (79 up-regulated and 33 down-regulated) for chromosome 1. The number of up-regulated genes was consistently higher than the number of down-regulated genes for all chromosomes except chromosome 13 (Figure 2).

Supplementary Table Legends

Supplementary Table 1: List of Differentially-Expressed Genes in Human Amniocytes From Fetuses with DS at an FDR Cut-off of 20%.

Supplementary Table 2: Gene Expression Profile of the Human HSA21 Orthologous Genes on the Mouse MMU16 in the Dp16, Ts65Dn and Ts1Cje Mouse Models of DS.

Supplementary Table 3: List of the Top 1% Up- and Down-Regulated Genes in Cell Types and Fetal Cerebrum and Cerebellum from Humans with DS.

Supplementary Table 4: DAVID Pathway Analyses Results in Cell Types and Brain Tissue from Humans with DS and Mouse Models.

Supplementary Table 5: Ingenuity Pathway Analyses (IPA) Results in Cell Types and Brain Tissue From Humans With DS and Mouse Models.

Supplementary Table 6: Gene Set Enrichment Analyses (GSEA/DFLAT) Results in Cell Types and Brain Tissue from Humans with DS and Mouse Models.

Supplementary Table 7: List of Differentially-Regulated Kinases and Phosphatases in Cell Types and Brain Tissue From Humans With DS.

Supplementary Figures Legends

Supplementary Figure 1: Primary Effects of Trisomy in Human Cell Types and Tissues From Humans with T21. Chromosome 21 differentially-regulated genes at FDR of 20% in multiple human cell types and tissues are represented. Genes consistently regulated in at least two human cell types and tissues are highlighted in blue. Genes differentially-expressed in all human tissues are indicated in pink.

Supplementary Figure 1: Primary Effects of Trisomy in Embryonic Day 15.5 Brains From Dp16, Ts65Dn and Ts1Cje Mouse Models of DS. Chromosome 16 orthologous genes differentially-regulated at FDR of 20% are represented. Genes consistently regulated in at least two mouse models are highlighted in blue. Genes differentially-expressed in all three mouse models are indicated in pink.

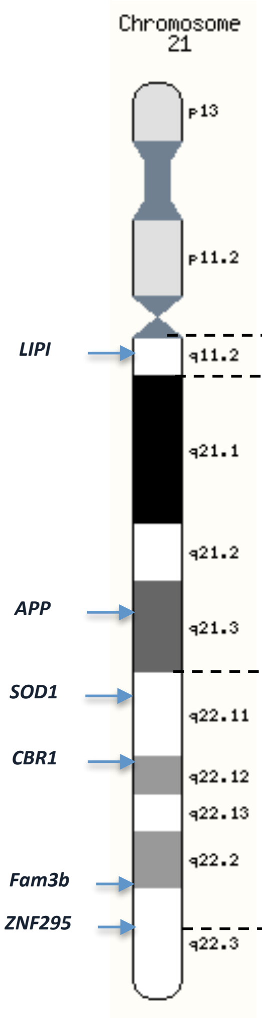

Band	Human Amniocytes and Amniotic Fluid	Human Fetal Cerebellum/ Cerebrum	Human iPSCs/Neurons
q11.2	AP001347.6, HSPA13	HSPA13, NRIP1	
q21.1	USP25, BTG3, C21ORF91, NCAM2	CXADR, BTG3, CHODL, NCAM2	USP25, CXADR
q21.2	D21S2088E		
q21.3	MIR155, MRPL39, ATP5J, RP1-100J12.1, USP16, CCT8, BACH1, MIR99AHG, LINC00307	ATP5J, APP, LTN1, USP16, CCT8	MRPL39, USP16, JAM2, ADAMTS1, N6AMT1, RWDD2B,
q22.11	TIAM1, SOD1, SCAF4, SYNJ1, IFNAR1, IFNAR2, GART, SON, DONSON, CRYZL1	SOD1, C21ORF59, TMEM50B, DONSON, CRYZL1, ATP5O	SCAF4, C21ORF59, PAXBP1, IL10RB, TMEM50B, GART, ITSN1, SMIM11
q22.12	CBR3, CHAF1B	DOPEY2, MORC3	SETD4
q22.13	RIPPLY3, TTC3, DSCR3	PIGP, TTC3	RIPPLY3, TTC3
q22.2	DYRK1A, ETS2, HMGN1, WRB<br>ZBTB21	PSMG1, WRB, PCP4	SH3BGR, IGSF5, PCP4
q22.3	U2AF1, RRP1B, PDXK, AGPAT3, C21ORF33, UBE2G2, C21ORF67, LOC642852, COL6A1, LSS, MCM3AP-AS1, YBEY, DIP2A, PRMT2, DSTNP1, C21ORF58	C2CD2, SLC37A1, PDE9A, PDXK, CSTB, SUMO3, PTTG1IP	TMPRSS2, LINC00479, TFF1, RSPH1, SLC37A1, PDE9A, CRYAA, H2BFS, CSTB, RRP1, PWP2, C21ORF33, PFLK, UBE2G2, FAM207A, COL18A1, SPATC1L, YBEY, PCNT, S100B

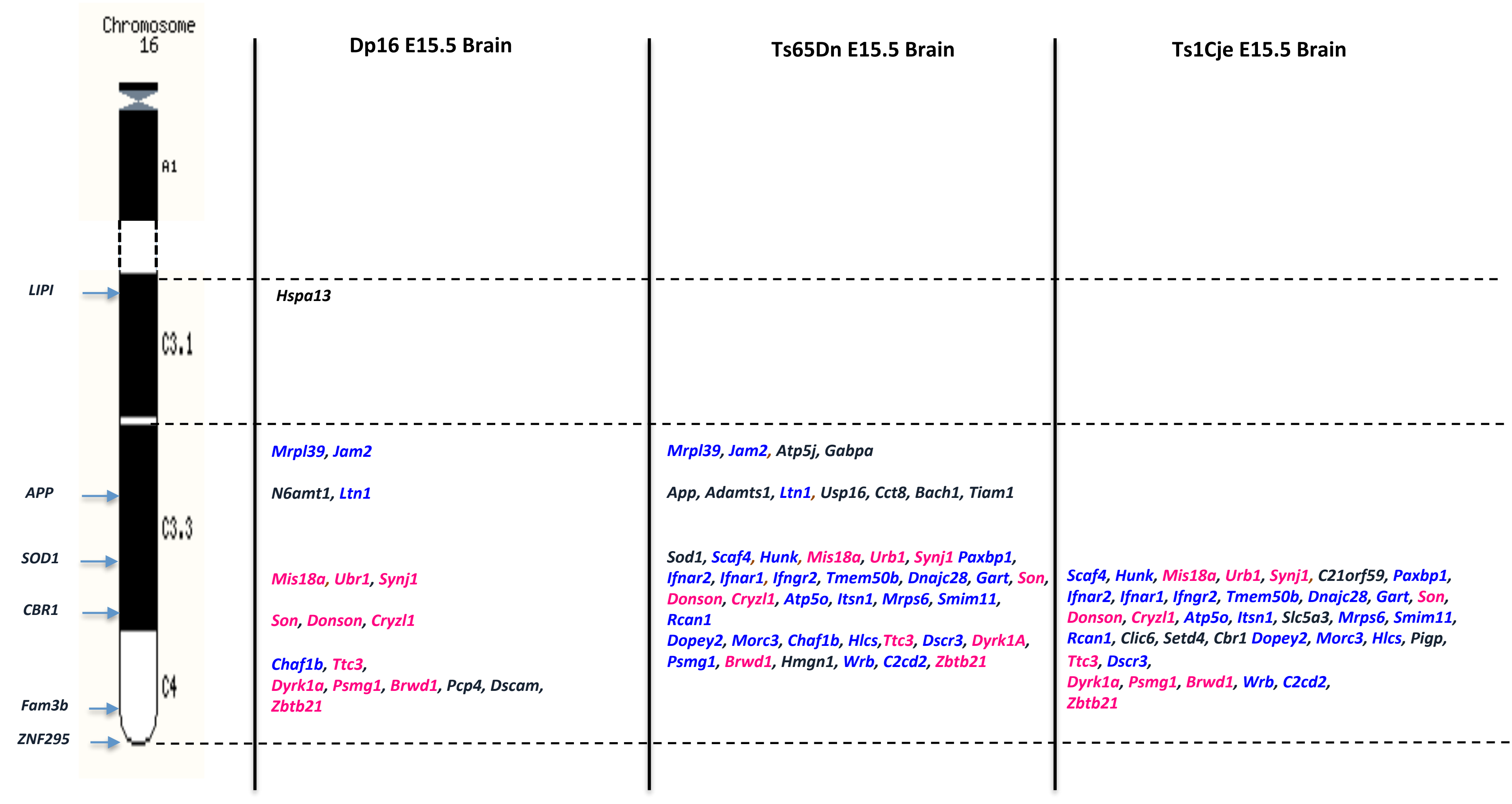

Chromosome 16
A1
C3.1
C3.3
C4
LIPI
APP
SOD1
CBR1
Fam3b
ZNF295
Dp16 E15.5 Brain
Ts65Dn E15.5 Brain
Ts1Cje E15.5 Brain
Hspa13
Mrpl39, Jam2
N6amt1, Ltn1
Mis18a, Ubr1, Synj1
Son, Donson, Cryzl1
Chaf1b, Ttc3,
Dyrk1a, Psmg1, Brwd1, Pcp4, Dscam,
Zbtb21
Mrpl39, Jam2, Atp5j, Gabpa
App, Adamts1, Ltn1, Usp16, Cct8, Bach1, Tiam1
Sod1, Scaf4, Hunk, Mis18a, Urb1, Synj1 Paxbp1,
Ifnar2, Ifnar1, Ifngr2, Tmem50b, Dnajc28, Gart, Son,
Donson, Cryzl1, Atp5o, Itsn1, Mrps6, Smim11,
Rcan1
Dopey2, Morc3, Chaf1b, Hlcs,Ttc3, Dscr3, Dyrk1A,
Psmg1, Brwd1, Hmgn1, Wrb, C2cd2, Zbtb21
Scaf4, Hunk, Mis18a, Urb1, Synj1, C21orf59, Paxbp1,
Ifnar2, Ifnar1, Ifngr2, Tmem50b, Dnajc28, Gart, Son,
Donson, Cryzl1, Atp5o, Itsn1, Slc5a3, Mrps6, Smim11,
Rcan1, Clic6, Setd4, Cbr1 Dopey2, Morc3, Hlcs, Pigp,
Ttc3, Dscr3,
Dyrk1a, Psmg1, Brwd1, Wrb, C2cd2,
Zbtb21
